# Glucosylation of (±)-Menthol by Uridine-Diphosphate-Sugar Dependent Glucosyltransferases from Plants

**DOI:** 10.3390/molecules26185511

**Published:** 2021-09-10

**Authors:** Elisabeth Kurze, Victoria Ruß, Nadia Syam, Isabelle Effenberger, Rafal Jonczyk, Jieren Liao, Chuankui Song, Thomas Hoffmann, Wilfried Schwab

**Affiliations:** 1 Biotechnology of Natural Products, School of Life Sciences, Technical University of Munich, Liesel-Beckmann-Str. 1, 85354 Freising, Germany; elisabeth.kurze@tum.de (E.K.); russ.victoria@web.de (V.R.); nadiasyam@hotmail.de (N.S.); rafjonde@gmail.com (R.J.); jieren.liao@tum.de (J.L.); tom.hoffmann@tum.de (T.H.); 2 4GENE, Lise-Meitner-Straße 30, 85354 Freising, Germany; isabelle.effenberger@4gene.de; 3 State Key Laboratory of Tea Plant Biology and Utilization, International Joint Laboratory on Tea Chemistry and Health Effects, Anhui Agricultural University, Hefei 230036, China; sckfriend@163.com

**Keywords:** *Camellia sinensis*, UDP-glucosyltransferase, UGT93Y1, menthol, menthyl glucoside

## Abstract

Menthol is a cyclic monoterpene alcohol of the essential oils of plants of the genus *Mentha*, which is in demand by various industries due to its diverse sensorial and physiological properties. However, its poor water solubility and its toxic effect limit possible applications. Glycosylation offers a solution as the binding of a sugar residue to small molecules increases their water solubility and stability, renders aroma components odorless and modifies bioactivity. In order to identify plant enzymes that catalyze this reaction, a glycosyltransferase library containing 57 uridine diphosphate sugar-dependent enzymes (UGTs) was screened with (±)-menthol. The identity of the products was confirmed by mass spectrometry and nuclear magnetic resonance spectroscopy. Five enzymes were able to form (±)-menthyl-β-d-glucopyranoside in whole-cell biotransformations: UGT93Y1, UGT93Y2, UGT85K11, UGT72B27 and UGT73B24. In vitro enzyme activity assays revealed highest catalytic activity for UGT93Y1 (7.6 nkat/mg) from *Camellia sinensis* towards menthol and its isomeric forms. Although UGT93Y2 shares 70% sequence identity with UGT93Y1, it was less efficient. Of the five enzymes, UGT93Y1 stood out because of its high in vivo and in vitro biotransformation rate. The identification of novel menthol glycosyltransferases from the tea plant opens new perspectives for the biotechnological production of menthyl glucoside.

## 1. Introduction

The natural product 2-isopropyl-5-methylcyclohexanol is a monocyclic monoterpene alcohol that has three asymmetric carbon atoms and, thus, occurs in eight stereoisomeric forms [1,2]. In addition to the enantiomers of menthol, three other pairs of diastereomers also called isomeric menthols exist, such as neomenthol, isomenthol and neoisomenthol [3]. The natural (−)-menthol is the most abundant isomer and is found in many essential oils, especially mint oils of the genus *Mentha* [4,5,6,7]. In Japanese peppermint oil from field mint (*Mentha arvensis*) grown in Japan or China, it accounts for up to 90% of the essential oil, and (−)-menthol is the main component in the peppermint oil (*Mentha piperita*). Menthols are also found in other genera and species of the Labiatae family, such as spearmint, and the spice plants basil (*Ocimum basilicum*), marjoram (*Origanum majorana*), oregano (*Origanum vulgare*), rosemary (*Rosmarinus officinalis*), sage (*Salvia* sp.) and thyme (*Thymus* sp.). The isomeric (+)-neomenthol is found in Japanese peppermint oil, while (−)-neoisomenthol makes up to one percent in geranium oil [3,4,7,8,9,10,11,12].

Over 19,000 metric tons of (−)-menthol are produced annually worldwide (http://www.leffingwell.com/menthol1/menthol1.htm; accessed on 8 September 2021), with about two-thirds derived from plants and one-third produced synthetically [7]. Several chemical synthesis routes have been established, including enantioselective production processes [4]. Plant-based extraction is accomplished by freezing out the crystalline menthol from the essential oil of *Mentha arvensis* [13]. At room temperature, menthol is a colorless, crystalline solid with a peppermint odor. The taste threshold is 0.4 ppm for (−)-menthol and 0.3 ppm for (+)-menthol (http://www.leffingwell.com/chirality/menthols.htm; accessed on 8 September 2021).

Menthol is added to a wide variety of products as a disinfectant and as a fragrance and flavoring agent, such as in confectionery and liquor, perfume, personal care, dental and oral hygiene products, lotions and hair lotions. Menthol is also used as an ingredient in skin care products [2,3,4,14].

Medicinally, menthol is a component of ointments (up to 6%) and liniments for the treatment of skin irritation caused by minor burns, insect bites or itching [2,3,15]. It acts at the cold menthol receptor (TRPM8) [16,17]. Therefore, menthol produces a cool sensation when applied to the skin but does not actually affect body temperature. When nasal cold receptors are irritated, the sensation of easier breathing is produced. In addition, menthol blocks the voltage-gated sodium channels, giving menthol a local anaesthetic effect. The activation of the GABA-A receptor explains the central depressant effect [3].

Due to its diverse physiological effects, menthol is in great demand and is highly valued by industry. The production of natural menthol requires significant areas of arable land and is dependent on climate. Given the volatility of mint crop prices due to variable crop yields and the environmental footprint of intensive mint cultivation, alternative bioproduction routes are being sought. Since the complete biosynthetic pathway in *Mentha* sp. has been known for some time, there are already the first attempts to transfer the biosynthetic route to host organisms by means of synthetic biology to increase production [7,18]. However, the low solubility of (−)-menthol in water, which is only 0.436 g L^−1^, and its toxicity impair the synthesis efficiency of microbial hosts and limits the usability of the monoterpene alcohol for a number of applications [2]. Since glycosylation significantly increases the water solubility of small molecules [19], various glycosylation strategies have been developed to form menthol conjugates. While menthyl glycosides were originally produced by modified Koenigs–Knorr reactions [20,21], biocatalytic processes were later developed for the production of water-soluble derivatives. Initially, transglycosylations and reverse hydrolysis reactions catalyzed by transglycosidases [22] and glycosidases [23,24,25,26], respectively, were employed, but recently the focus has been on uridine diphosphate sugar-dependent glycosyltransferases (UGTs) because these enzymes catalyze the glycosylation of small molecules in cells [14,27,28]. UGTs transfer the sugar moiety of an activated nucleotide-diphosphate (NDP) carbohydrate to an acceptor molecule to provide a thermodynamically favoured *O*-glycoside, *C*-glycoside, *N*-glycoside or *S*-glycoside product [19]. The main driving force for the glycosylation reaction to proceed to completion is the exergonic release of NDP from the sugar donors. UGTs are capable of glycosylating a variety of acceptor molecules with high regioselectivity and enantioselectivity, which is why protective groups are not required [29].

More than 800,000 glycosyltransferases, subdivided into 114 families, have been identified according to the carbohydrate active enzymes (CAZy) database (http://www.cazy.org/) (retrieved 25 June 2021), with family 1 among those containing the most members [30]. Glycosyltransferases are classified according to four different properties, namely class of substrates, protein structure, preferred stereochemistry and dependence on metals for catalytic activity [29]. The proteins can be distinguished because of their different folds, with GT-A and GT-B folds predominating. Both folds contain separate donor and acceptor binding sites. Glycosyltransferase family 1 includes NDP-dependent enzymes whose sugar donor binding site is located in the C-terminal part of the proteins. The corresponding representatives from plants preferentially glycosylate small molecules and prefer UDP-activated sugars as donor molecules. The plant secondary product glycosyltransferase (PSPG) box was identified as the sugar donor interaction site in these enzymes [31].

Due to the versatility of plant UGTs, the detection of (−)-menthyl glucoside in plants [10,11,27,32,33,34,35,36] and the great interest in efficient biocatalysts for the glycosylation of (±)-menthol, we screened a subset of our UGT library, which includes more than 100 enzymes, with (±)-menthol by whole-cell biotransformation. The method used has already been successfully applied for the identification of furanone UGTs [37]. Five enzymes were identified, functionally characterized and used for the production of (±)-menthyl glucoside in a bioreactor.

## 2. Results

### 2.1. Cloning and Expression of UGT93Y1 and UGT93Y2

To add additional members to an already existing UGT library, putative UGT sequences in *Camellia sinensis* var. *sinensis* were searched by BLAST search in the Tea Plant Information Archive database (http://tpia.teaplant.org; accessed on 8 September 2021). The coding sequences of the full-length candidate genes TEA009739 and TEA009753 were randomly selected. Their cDNA sequences were obtained from the leaves of the tea plant *C. sinensis* var. *sinensis*, cloned into the pGEX-4T1-vector and the encoded UGTs were successfully produced as N-terminal glutathione S-transferase (GST) fusion proteins in *Escherichia coli* BL21(DE3)pLysS. After affinity purification and verification by SDS-PAGE, the analyses showed clear bands for the fusion proteins at around 80 kDa (Supplementary material Figure S1). In addition, a band of about 27 kDa was visible for the GST, and few bands showed co-purified proteins. The production of UGT-GST fusion proteins was confirmed by Western blot analysis by using a monoclonal anti-GST antibody (Supplementary material Figure S1). The encoded proteins were subsequently assigned UGT93Y1 and UGT93Y2 by the UGT Nomenclature Committee. *UGT93Y1* and *UGT93Y2* are 1404 and 1413 base pairs long, respectively, encode proteins with a length of 467 and 470 amino acids, respectively, and share a sequence identity of 74.3%. Both sequences show the characteristic features of functional UGTs of the CAZy family 1, including the catalytically active amino acids histidine at position 29 [38], the activating aspartic acid (position 134) and the 44 amino acid long PSPG box [19,31] (Supplementary material Figure S2). The GSS motif, a feature of mono-glucosyltransferases [39], is replaced with TSS at position 413–415 (UGT93Y1) and ASS at position 416–418 (UGT93Y2). Eventually, *Escherichia coli* Waksman cells were transformed with the respective plasmid carrying *UGT93Y1* or *UGT93Y2* for whole-cell biotransformation.

### 2.2. In Vivo Substrate Screening Using E. coli Waksman

**Figure 1:**
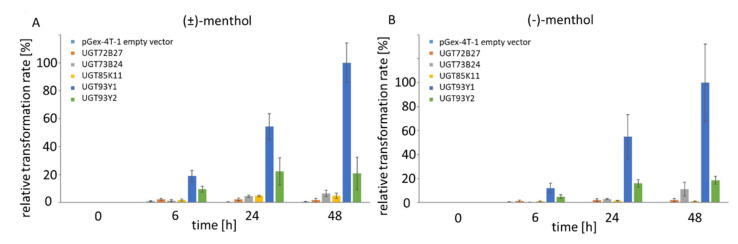
Relative biotransformation rate of UGT72B27, UGT73B24, UGT85K11, UGT93Y1 and UGT93Y2 in % towards (±)-menthol (**A**) and (−)-menthol (**B**). The intensity of the pseudo-molecular ion *m/z* 363 [M + HCOO]^−^ of menthyl glucoside was determined, and the UGT with the highest value was set to 100%. Samples were analyzed after 6, 24 and 48 h after substrate addition. pGEX-4T-1 served as negative control.

*Escherichia coli* Waksman cells expressing recombinant plant UGTs were employed as whole-cell biocatalysts in order to identify (±)-menthol UGTs, similar to the method described in [37]. Menthyl glucoside produced by the biocatalysts was identified by LC-MS (Supplementary material Figure S3). By comparing the intensities of the signals for the pseudo-molecular ion *m/z* 363 [M + HCOO]^−^ of (±)-menthyl glucoside, the relative biotransformation activities of the biocatalysts were calculated, with the highest value set at 100% (Supplementary material Figure S4). The sreening of a library of family 1 UGTs containing enzymes from *Arabidopsis thaliana*, *C. sinensis*, *Catharanthus roseus*, *Fragaria x ananassa*, *Fragaria x vesca*, *Nicotiana benthamiana*, *Rubus idaeus*, *Starmerella bombicola* and *Vitis vinifera* revealed that only 5 out of 57 UGTs were able to glucosylate (±)-menthol in vivo (Supplementary material Figure S4). The most efficient biocatalyst was the strain expressing the newly identified UGT93Y1, followed by UGT85K11 and UGT93Y2. All three enzymes originated from the tea plant *C. sinensis*: UGT72B27 from *V. vinifera* and UGT73B24 from *F.* x *ananassa* glucosylate menthol to a minor extent. The (±)-menthyl glucoside was not produced by the other 52 UGTs after 48 h of biotransformation. In order to test the stereospecificity of the five biocatalysts, UGTs were incubated individually with (±)-menthol and (−)-menthol (Figure 1). As early as six hours after substrate addition, the corresponding menthyl glucosides were already detected in the cell supernatant, and their concentration increased for at least up to 48 h. Similar biotransformation rates were calculated after the addition of racemic and (−)-menthol, suggesting that the enzymes are not stereospecific for menthol enantiomers.

### 2.3. Qualitative Substrate Screening of UGT93Y1 and UGT93Y2 by LC-MS

**Figure 2:**
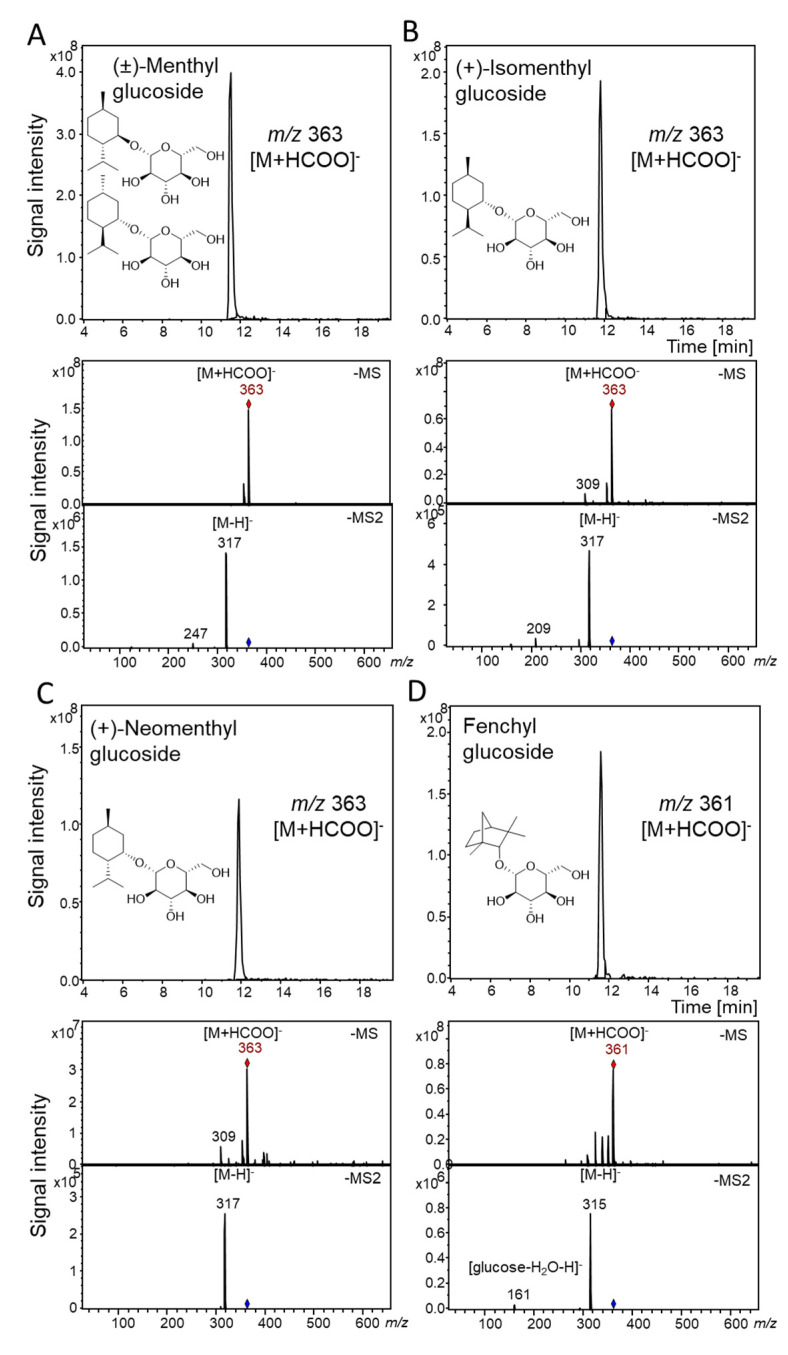
Qualitative in vitro substrate screening of UGT93Y1 with (±)-menthol (**A**), (1*S*,2*R*,5*R*)-(+)-isomenthol (**B**), (1*S*,2*S*,5*R*)-(+)-neomenthol (**C**) and fenchyl alcohol (**D**) by LC-MS. Selected ion chromatograms (*m/z* 363 and 361), mass spectra in negative mode (-MS) and product ion spectra of the pseudo-molecular ions (*m/z* 363 and 361; -MS2) are shown.

Since the two recombinant UGTs identified in *C. sinensis* var. *sinensis* (UGT93Y1 and UGT93Y2) efficiently glucosylated menthol, they were analyzed in more detail. Substrate screening was performed by incubating the purified protein extract of UGT93Y1 and UGT93Y2 with the sugar donor UDP-glucose and various potential acceptor (monoterpenol) substrates. The reaction products of the glycosyltransferase reaction were analysed by LC-MS, and glucosides were identified by their MS and MS2 spectra, relative retention time and by comparison with the spectral data of authentic reference material. UGT93Y1 revealed superior enzymatic activity towards the monocyclic monoterpenoid (±)-menthol, (Figure 2A). Moreover, the enzyme was able to glucosylate the menthol isomers (+)-isomenthol and (+)-neomenthol, as well as the bicyclic monoterpenoid fenchyl alcohol (Figure 2B–D). The three glucosides of the menthol isomers showed corresponding pseudo-molecular ions for the glucoside product at *m/z* 363 [M + HCOO]^−^, and fenchyl glucoside was detected by means of the pseudo-molecular ion *m/z* 361 [M + HCOO]^−^. Despite the high amino acid sequence similarity, UGT93Y2 glycosylated menthol only to a lesser extend (Supplementary material Figure S5). Therefore, the following quantitative substrate screening using UDP Glo™ Glycosyltransferase assay was performed with UGT93Y1 only.

### 2.4. UDP Glo™ Glycosyltransferase Assay

**Figure 3:**
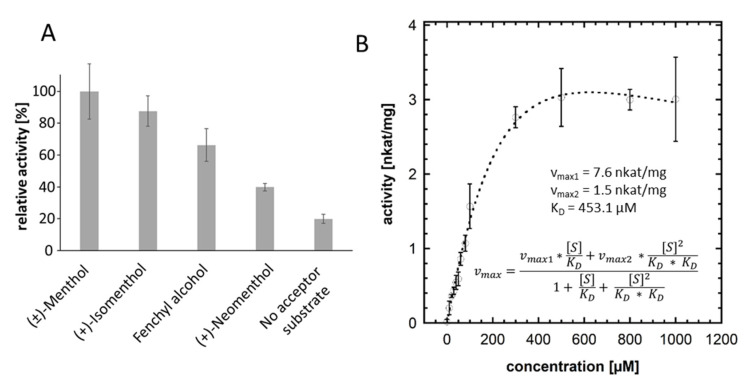
Determination of the relative enzymatic activities with UDP Glo™ Glycosyltransferase assay (**A**) and UGT93Y1 enzyme kinetics for (±)-menthol (**B**). The relative enzyme activity was calculated after measuring the release of UDP during the glucosylation reaction. The highest enzymatic activity was set at 100%, and the relative enzyme activities calculated (**A**). The enzyme activity versus substrate concentration graph for UGT93Y1 from *C. sinensis* var. *sinensis* was determined using the UDP Glo™ Glycosyltransferase assay. The calculation was performed with KaleidaGraph (https://www.synergy.com/; v4.5.4; accessed on 8 September 2021).

In order to characterize the enzyme activity of UGT93Y1 in more detail with respect to its particular substrate specificity towards menthol, a quantitative substrate screening was performed using the UDP Glo™ Glycosyltransferase assay [40]. In this assay, the enzymatically released amount of UDP is determined to calculate relative activities. The preferred substrate for UGT93Y1 was (±)-menthol (100% corresponding to 0.11 nkat mg^−1^), whereby the enzyme activity towards the two isomeric forms was lower (Figure 3A). In addition, the recombinant UGT93Y1 showed UDP-α-D-glucose hydrolase activity when no acceptor substrate was added to the enzyme reaction as a low amount of UDP was detectable. The glycosyltransferase reaction was optimized with respect to protein amount, incubation time, incubation temperature and pH, and the enzyme kinetics for UGT93Y1 were determined using 0 to 1000 µM (±)-menthol (Figure 3B). The enzyme activity versus substrate concentration graph shows a substrate inhibition curve [41]. Calculations of the kinetic data assuming a two-site sequential enzyme model with identical dissociation constants produced 7.6 and 1.5 nkat/mg for the maximum reaction velocities v_max1_ and v_max2_, respectively, while the dissociation constant K_D_ was 453.1 µM.

The in vitro enzyme activity assays by qualitative LC-MS analysis (Figure 2), quantitative substrate screening (Figure 3A) and kinetic analysis performed by UDP Glo™ Glycosyltransferase assay (Figure 3B) as well as in vivo experiments using *E. coli* Waksman cells (Figure 1) revealed high glycosyltransferase activity of UGT93Y1 towards menthol, resulting in the formation of menthyl glucoside.

### 2.5. Characterization of Menthyl Glucoside by NMR

**Figure 4:**
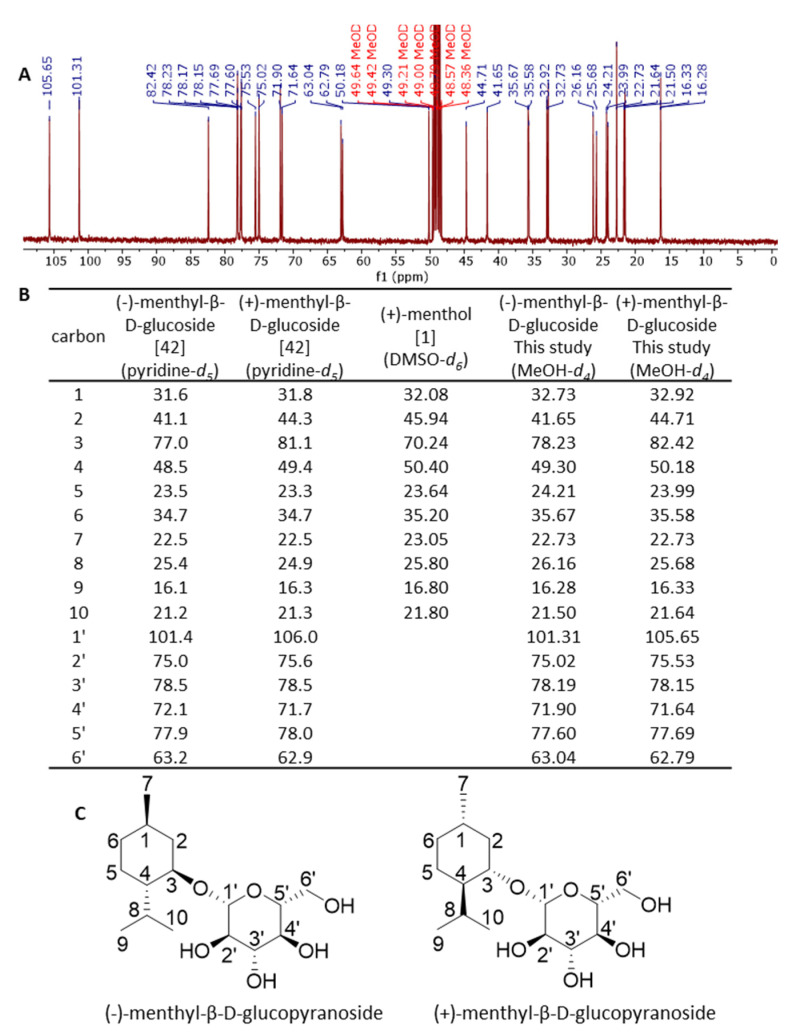
^13^C NMR analysis of biotransformation products formed from (±)-menthol by UGT93Y1 whole-cell biocatalysis. NMR spectrum of products produced from (±)-menthol (**A**). Chemical shifts (δ) in ppm of (−)- and (+)-menthyl-β-d-glucopyranoside taken from [42] of (+)-menthol from [1] and of the reaction products (**B**). Chemical structures of (−)- and (+)-menthyl-β-d-glucopyranoside (**C**).

The biotransformation product of (±)-menthol was produced on a larger scale and isolated by solid phase extraction. The structures of the glucosides were analyzed by NMR spectroscopy (Figure 4). The ^1^H-NMR and ^13^C-NMR signals of the products were assigned by studies of the ^1^H-^1^H COSY, HMQC and HMBC spectra, as well as reference data from the literature [14,32,34,42,43,44]. The ^1^H-NMR spectra (400 MHz, MeOD) of the glucosylated racemic menthol showed the characteristic signals due to β-d-glucopyranosyl residues of δ 4.38 ppm (d, *J* = 7.8 Hz, 1H) for the (−)-menthyl conjugate and of δ 4.34 ppm (d, *J* = 7.7 Hz, 1H) for the (+)-menthyl derivative. The ^13^C-NMR spectra exhibited signals for the anomeric carbons at 101.31 and 105.65 ppm (C1’) for (−)- and (+)-menthyl β-d-glucoside, respectively (Figure 4). These signals reflect the production of two diastereomers from racemic menthol by the conjunction with β-d-glucopyranose. Splitted signals were also observed for all carbons. The signals for the anomeric 1’ carbons showed almost the same intensity. Based on the NMR and literature data, the biotransformation products formed by the UGT93Y1 whole-cell biocatalyst were established to be (±)-menthyl β-d-glucopyranosides. Since the racemic acceptor substrate was added in excess, the results imply that UGT93AY1 shows low stereospecificity for (±)-menthol (Figure 4).

### 2.6. Homology Modeling and Ligand Docking

**Figure 5:**
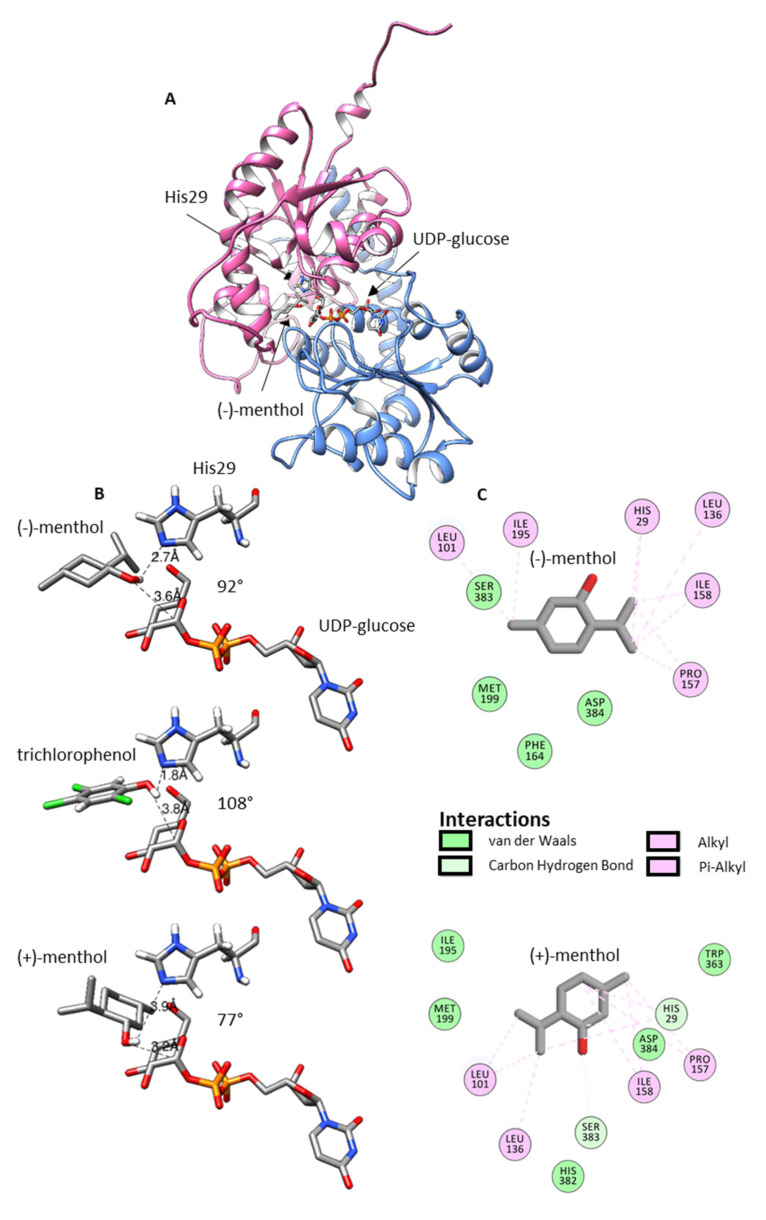
Homology modelling. Prediction of the 3D structure of UGT93Y1 was performed by the IntFOLD Integrated Protein Structure and Function Prediction Server (https://www.reading.ac.uk/bioinf/IntFOLD/; accessed on 8 September 2021) with default values. The result was visualized with UCSF Chimera (https://www.cgl.ucsf.edu/chimera/; accessed on 8 September 2021), and ligand docking was performed with the Autodock/Vina tool (http://vina.scripps.edu/download.html; accessed on 8 September 2021). Predicted 3D structure of UGT93Y1 (**A**), predicted (−)-menthol and (+)-menthol pose in the active site of UGT93Y1 (trichlorophenol pose from 2vce (https://www.rcsb.org/; accessed on 8 September 2021) is shown for comparison) (**B**) and ligand–protein interactions predicted by Discovery Studio Visualizer v19.1.0.18287 (https://discover.3ds.com/discovery-studio-visualizer-download; accessed on 8 September 2021) (**C**).

To better understand the structural requirements of enzymatic glucosylation of racemic menthol, homology modelling was performed. Therefore, the amino acid sequence of UGT93Y1 was submitted to IntFOLD web server (https://www.reading.ac.uk/bioinf/IntFOLD/; accessed on 8 September 2021). Among the top five 3D models, model IntFOLD6HHpredTS5 was chosen due to its high GMQS of 0.6541 and confidence *p*-value of 2.293 × 10^−14^. Scores greater than 0.4 are characteristic for more complete and confident models; thus, the predicted model is very close to the native protein 3D structure [45]. With the exception of the first 10 amino acids at the N-terminus, no residue exceeded the disorder/order probability score of 0.5 (Supplementary material Figure S6), while the conserved regions in UGTs (catalytic His29, activating Asp134 and the PSPG box from Trp342 to Gln385) are located in highly ordered regions of the protein. Accordingly, these residues and regions also show low per-residue errors (Supplementary material Figure S7). UGT93Y1 is a typical GT1 family member; it adopts the GT-B fold, and it should follow an inverting mechanism (Figure 5).

Ligand ((−)-menthol and (+)-menthol) docking was performed on the predicted 3D model of UGT93Y1 by using the Autodock Vina tool implemented in UCSF Chimera and the protein 3D structure 2vce (https://www.rcsb.org/; accessed on 8 September 2021) with the acceptor and donor ligands trichlorophenol and UDP-glucose, respectively, as template. The ligand (−)-menthol was installed in chair conformation with equatorial arranged substituents in a plane with trichlorophenol in the active site (Figure 5). The bond lengths (2.7 and 3.6 Å) and angle (92°) of the arrangement His29/(−)-menthol-OH/C1-UDP-glucose correspond well to the lengths (1.8 and 3.8 Å) and angle (108°) of the catalytic conformation in the crystal structure of 2vce. In the case of the (+)-menthol enantiomer, the structure is rotated horizontally by 180° (the methyl group now facing the catalytically active His29) and tilted a few degrees to the side. This extends the bond lengths (3.9 and 3.2 Å) and reduces the angle to 77° and might explain the slight preference of UGT93Y1 for (−)-menthol. A closer look at the active site shows that it is lined with nonpolar amino acids (Leu101, Leu136, Pro157, Ile158, Phe164, Ile195 and Met199), which presumably interact polar (van der Waals and carbon hydrogen) and nonpolar (alkyl and Pi-alkyl) with terpene alcohol (Figure 5) similar to the diterpene steviol glucoside in UGT76G1 [46].

## 3. Discussion

In this study, we identified five UDP-glucose-dependent glycosyltransferases (UGT93Y1, UGT93Y2, UGT85K11, UGT72B27 and UGT73B24) by screening 57 members of a UGT whole-cell library that were capable of glucosylating (±)-menthol in vivo and in vitro (Supplementary material Figure S4 and Figure 1). Two new plant biocatalysts from *C. sinensis* (UGT93Y1 and UGT93Y2) showed outstanding enzymatic activities compared to the other catalytically active proteins. These can be used for the development and improvement of biotechnological processes for the production of small molecule glucosides, which are widely used for industrial applications in pharmaceutical, food or cosmetics industries. Due to its minty smell, cooling and refreshing properties, menthol is widely applied as a flavor and fragrance additive in different industries [14]. Glycosylation using enzymes that have major benefits compared to chemical synthesis can increase the low water solubility of 435.5 mg L^−1^ at 25 °C [2] of this physiologically active compound, which impedes various applications [31]. Recombinant plant glycosyltransferases from *Arabidopsis thaliana* have already been described to glucosylate monoterpenoids such as geraniol, nerol, linalool and terpineol including (±)-menthol. [47]. However, only glycosyltransferases of the UGT73C clade (C1, C3, C5 and C6) were identified that could convert (±)-menthol. Here, we show that UGTs of the 93, 85 and 72 clades are also able to metabolize the monoterpenol in addition to UGT73B24. A number of UGT85 members have already been identified as monoterpene UGTs [48], while for the UGT72 group no enzyme has been detected so far that converts monoterpene alcohols. Thus, it is comprehensible that, in our study, the UGT72B27 protein showed the lowest activity. UGT93Y1 and UGT93Y2 are the new members of the 93 group and form a new subgroup of their own. UGT93Y1 shows an extraordinary menthol glycosylation activity, which is unusual, as the plant from which the corresponding *UGT93Y1* gene was isolated from does not produce menthol. This result confirms once more that some UGTs involved in secondary plant metabolism show broad substrate tolerance [48], which is underpinned by the activity towards other menthol isomers such as (+)-isomenthol and (+)-neomenthol and the bicyclic (±)-fenchyl alcohol (Figure 2).

In addition to its menthol glucosyltransferase activity, UGT93Y1 exhibits UDP-α-D-glucose glucohydrolase activity (Figure 3A), which has already been demonstrated for a number of glycosyltransferases [49,50,51]. This means that the enzyme is able to transfer the glucose molecule from UDP-glucose to the acceptor water in the absence of other acceptor substrates. This results in the release of UDP from the sugar donor, which can be detected by the UDP Glo™ glycosyltransferase assay. Fortunately, this background reaction has been previously observed with many UGTs and can be exploited to determine sugar donor specificity [50]. However, the biological relevance is unknown.

Even though UGT93Y1 and UGT93Y2 show high sequence identity (74.3%), both enzymes differ significantly in their ability to metabolize (±)-menthol (Figure 1, Supplementary material Figures S4 and S5). Inspection of the amino acids lining the active site (Figure 5) shows that three of them (I158T; I195V and M199F; Supplementary material Figure S2) are exchanged in the catalytic center of UGT93Y2 compared with UGT93Y1. While position 158 is located in a region of higher disorder (Supplementary material Figure S6) and higher structural inaccuracy (Supplementary material Figure S7), positions 195 and 199 are located in regions of lower disorder and higher prediction accuracy. These positions are likely to be essential for activity toward (±)-menthol and could be targeted by site-directed mutagenesis [52] or error-prone PCR [53] to find variants with increased catalytic efficiency.

UGT93Y1 shows non-Michaelis–Menten kinetics as the enzyme activity is inhibited at high substrate concentrations (Figure 3B). Substrate inhibition is a well-known feature of human glucuronosyltransferases [54] and has also been described for plant glycosyltransferases [55]. The significance of such "atypical" kinetics in vivo is not yet understood but is observed for many enzymes [56]. In recent years, it has become apparent that the mechanisms of substrate inhibition are very complex. Multiple (at least two) binding sites within the enzyme, the formation of a ternary dead-end enzyme complex and/or ligand-induced changes in enzyme conformation, among others, are discussed as causes of substrate inhibition [54]. Substrate inhibition is undesirable in biotechnological processes, but studies have shown that inhibition of the catalytic activity of enzymes by an excess of substrate can be overcome by replacing individual amino acids in the protein [57].

According to the NMR and literature data, the products formed by UGT93Y1 during the whole-cell biotransformation were established to be (±)-menthyl β-d-glucopyranosides. Thus, UGT93Y1 isolated from *C. sinensis* does not stereospecifically glucosylate racemic menthol, which could be one reason for its high activity. Plants appear to be a rich source of promiscuous UGTs that not only glycosylate substrates of their native host but also non-native acceptor molecules. Similarly, *Eucalyptus perriniana* cells metabolize (−)-and (+)-menthol, a non-native metabolite of this plant, to the respective monoterpenyl 3-*O*-β-d-gentiobioside and 3-*O*-β-d-triglucoside (2,6-di-*O*-(β-d-glucopyranosyl)-β-d-glucopyranoside), respectively [34,58]. Moreover, in this plant, the first two glucose transfers appear to be nonspecific, whereas the third glucose unit is transferred only to the (+)-menthyl derivative.

In contrast to plant cell cultures, whole-cell biotransformation using *E. coli* cells represents a simple but highly efficient alternative for the production and purification of glucosides [59,60,61]. With minor improvements in cultivation parameters, an economical large-scale bioprocess can be developed, replacing expensive chemical synthesis that requires protection and deprotection reactions, resulting in low specificities and yields. Various terpene glycosides occur naturally and are obtained in small amounts from plant extracts [48,62,63]. Due to their high economic importance, glycosylation using enzyme biocatalysts becomes very attractive for the synthesis of glycosides and exhibits high efficiency, stereoselectivity and regioselectivity in an environmentally friendly manner.

## 4. Materials and Methods

### 4.1. Chemicals

All chemicals and reagents were purchased in analytical scale from Sigma Aldrich, Steinheim, Germany, unless otherwise mentioned.

### 4.2. RNA Isolation, cDNA Synthesis and Cloning of UGT93Y1 and UGT93Y2

Total RNA form tea leaves of *C. sinensis* var. *sinensis* were isolated according to the CTAB method [64] with minor modifications. The extraction buffer was prepared without spermidine. After precipitation overnight at 4 °C, the RNA pellet was solved in 600 µL RLT Plus Buffer of the RNeasy^®^ Plant Mini Kit from Qiagen. The following steps were performed according to manufacturer’s instructions. The RNA concentration was determined using the CLARIOstar LVis plate (BMG Labtech, Ortenberg, Germany). The integrity of the RNA preparation was confirmed by agarose gel electrophoresis.

First strand cDNA synthesis using the M-MLV Reverse Transcriptase was performed according to the manufacturer’s instructions (Promega, Mannheim, Germany).

The transcribed cDNA was used as a template for PCR to amplify the open reading frame (ORF) sequence of the *UGT93Y1* (http://tpia.teaplant.org, TEA009739; accessed on 8 September 2021) and *UGT93Y2* (http://tpia.teaplant.org, TEA009753; accessed on 8 September 2021) genes using gene-specific primers introducing enzyme restriction sites (fw_UGT93Y1_*BamH*I CGGGAT-CCATGGATGTTCCTAACCAACAT; rev_UGT93Y1_*Xho*I CCCTCGAGCTACCTAGTTATGTGAGCAAT; fw_UGT93Y2_*BamH*I CGGGATCCATGGATGTTCATGACCAACAT; rev_UGT93Y2_*Xho*I CCCTCGAGCTACCGAGT TATGTGAGCAAT). After gel extraction of the correct DNA fragments with the PCR Clean-up Gel Extraction Kit (Macherey-Nagel, Düren, Germany), PCR products were cloned into pGEM^®^-T E vector system via A-tailing according to the manufacturer’s instruction (Promega, Mannheim, Germany). The resulting plasmid construct was digested with *BamH*I and *Xho*I and ligated into the predigested pGEX-4T1 vector. After verification of the sequences by Genewiz (Leipzig, Germany), pGEX-4T1 plasmid constructs were transformed in *Escherichia coli* BL21 (DE3)pLysS (Novagen, Darmstadt, Germany).

### 4.3. Recombinant Protein Production and Protein Purification

Protein expression was performed using *E*. *coli* BL21(DE3)pLysS cells containing pGEX-4T-1 *UGT93Y1* and pGEX-4T-1 *UGT93Y2* according to [65], with minor modifications. After growing the pre-cultures overnight at 37 °C and 150 rpm in Luria-Bertani medium containing 100 µg/mL ampicillin and 34 µg/mL chloramphenicol, 1 L main culture and containing the corresponding antibiotics, it was inoculated with 10 mL of pre-culture and further incubated at 37 °C and 120 rpm until OD_600_ reached 1 in a chicane flask. Gene expression was induced with 1 mM isopropyl-β-d-thiogalactopyranoside, and cultures were incubated over night at 18 °C at 150 rpm. Cells were harvested via centrifugation, and the pellets were stored at −80 °C.

Recombinant fusion proteins with an N-terminal GST tag were purified by Novagen^®^ GST Bind™ Resin following the manufacturer’s instructions. The cells were resuspended in the binding buffer and disrupted by sonication. The crude protein extract was incubated over night at 4 °C with the resin to bind GST fusion protein. The recombinant protein was eluted with GST elution buffer containing reduced glutathione. The quality and quantity of the purified proteins was verified using SDS-PAGE, and the protein concentration was determined with Roti^®^Nanoquant (Carl Roth, Karlsruhe, Germany).

### 4.4. Qualitative Enzyme Assay Using LC-MS Screening

For the initial substrate screening, the glycosyltransferase reaction was performed according to [39] with minor modifications. The enzyme reaction with a final volume of 100 µL containing 5 µg of purified recombinant protein in 100 mM TRIS–HCl buffer (pH 7.5), 1 mM UDP-glucose and 600 µM substrate dissolved in dimethyl sulphoxide (DMSO). The reaction was incubated at 30 °C, with constant shaking at 400 rpm overnight. LC-MS analysis was performed according to literature [39].

### 4.5. Quantitative UDP Glo™ Glycosyltransferase Screening

Positive substrates that were determined via LC-MS were further screened via UDP Glo™ Glycosyltransferase Assay (Promega, Mannheim, Germany) according to the manufacturer’s instruction in order to determine the kinetic parameters of the UGTs. The enzymatic reaction was performed according to [66] with minor modifications. After starting the reaction by adding UDP-glucose, samples were incubated for 30 min at 30 °C and 400 rpm. The reaction was stopped by adding 12.5 µL 0.6 M HCl and further neutralization with 1 M TRIZMA base. Five µL of the GT reaction was pipetted to a 384 well plate [40]. The calculation of kinetic data was performed with KaleidaGraph (https://www.synergy.com/; v4.5.4; accessed on 8 September 2021).

### 4.6. Screening of UGT-Library for Whole-Cell Biotransformation

A library of 57 plant UGTs from *Arabidopsis thaliana*, *C. sinensis*, *Catharanthus roseus*, *Fragaria x ananassa*, *Fragaria x vesca*, *Nicotiana benthamiana*, *Rubus idaeus*, *Starmerella bombicola* and *Vitis vinifera* was screened for the glucosylation of menthol: AtUGT73C5, UGT71A33, UGT71A34a, UGT71A35, UGT71A44, UGT71AJ1, UGT71K3a, UGT71K3b, UGT71W2, UGT72AY1, UGT72B1, UGT72B27, UGT730A1, UGT73A15, UGT73AR2, UGT73B23, UGT73B24, UGT73E12, UGT73E13, UGT73E14, UGT73E15, UGT73E16, UGT75L22, UGT75L23, UGT75L24, UGT75T1, UGT76Q2, UGT76Q3, UGT84A41, UGT84A42, UGT84A43, UGT84A44, UGT84A45, UGT84A46, UGT84A47, UGT84A48, UGT84A49, UGT85A69, UGT85A69co, UGT85A70, UGT85A72, UGT85A72, UGT85K11, UGT88A12, UGT88A20, UGT88A21, UGT88A22, UGT88A23, UGT88A24, UGT88A25, UGT88A26, UGT88A27, UGT88A27, UGT88F12, UGT92G6, UGT93Y1 and UGT93Y2 (http://prime.vetmed.wsu.edu/resources/udp-glucuronsyltransferase-homepage; accessed on 8 September 2021).

The corresponding genes were cloned into pGEX-4T-1 vector, and *E*. *coli* Waksman cells were transformed with the respective plasmid. The enzyme activity of various UGTs towards (±)-menthol was investigated in a small-scale biotransformation process according to [37]. The in vivo biotransformation begun by adding 0.5 mg/mL (±)-menthol at 18 °C. The samples were analyzed in triplicates, and the production of menthyl glucoside was investigated 6, 24 and 48 h after substrate addition. The culture supernatant was centrifuged twice (13.500 rpm, RT and 10 min). Menthyl glucoside formation was analyzed via LC-UV-MS. For relative quantification, signals for *m/z* 363 (M+HCOO-; formic acid adduct) were integrated. The highest value was set to 100%, and the relative biotransformation rate was calculated.

### 4.7. Production and Purification of Menthyl Glucoside from Large Scale Biotransformation

Menthyl glucoside was produced by whole-cell biotransformation on a large-scale in a 5 L fermenter batch according to [37]. In vivo biotransformation was induced by adding (±)-menthol (Sigma Aldrich, Steinheim, Germany) to the bacterial culture. Purification of the glucoside was performed as described in [37].

### 4.8. NMR Analysis

Thirty-five mg of purified glucoside was solved in 200 µL methanol-*d_4_* (Sigma-Aldrich, Steinheim, Germany), centrifuged at maximum speed for 2 min and evaporated. The residue was solved in 400 µL methanol-*d_4_* (Sigma-Aldrich, Steinheim, Germany), transferred to an NMR tube and filled up to a final volume of 600 µL. NMR spectra were recorded with a Bruker DRX 500 spectrometer (Bruker, Karlsruhe, Germany). The chemical shifts were referred to the solvent signal. The one-dimensional and two-dimensional COSY, HMQC and HMBC spectra were acquired and processed with standard Bruker software (XWIN-NMR) and MestreNova software (mestrelab.com; accessed on 8 September 2021).

### 4.9. Homology Modelling of UGT93Y1 and Molecular Docking

The protein sequence of UGT93Y1 was submitted to IntFOLD web server (https://www.reading.ac.uk/bioinf/IntFOLD/; accessed on 8 September 2021) in order to develop a model with sufficient query sequence coverage and sequence identity [45]. The most reliable 3D structure was selected based on the Global Model Quality Score (GMQS 0.6541) and confidence *p*-value (2.293 × 10^−14^). The GMQS ranged between 0 and 1, and scores greater than 0.4 generally indicate more complete and confident models, which are highly similar to the native structure. The consistency of the GMQS allows calculating a *p*-value, which quantifies the probability that a model is incorrect. The *p*-value = 2.293 × 10^−14^ means that 1/2.293 E14 incorrect models will have higher scores. Templates used for homology modelling were 2vch_A, 2acv_A, 2c1x_A, 2pq6_A and 5tme_A (https://www.rcsb.org/; accessed on 8 September 2021). The docking of (−)-menthol and (+)-menthol in the active site of UGT93AY1 was performed with the Autodock/Vina tool [67] implemented in UCSF Chimera (https://www.cgl.ucsf.edu/chimera/download.html; accessed on 8 September 2021).

## Data Availability

Not applicable.

## Supplementary Materials

The following are available online. Figure S1: SDS-PAGE (A) and Western blot (B) using a primary monoclonal Anti-Glutathione-S-Transferase (GST) antibody produced in mouse (Sigma Aldrich) of recombinant UGT93Y1 (1) and UGT93Y2 (2) in purified protein elution fractions. Blue and green arrows show the UGTs and GST proteins, respectively. Marker proteins (M), Figure S2: Amino acid sequences of UGT93Y1 and UGT93Y2 from *C. sinensis* var. *sinensis*. The catalytically active His and the activating Asp are marked with red and blue arrows, respectively. The red box represents the conserved PSPG box, Figure S3: LC-MS analysis of (±)-menthyl glucoside Extracted ion chromatogram *m/z* 363 [M + HCOO]^−^ (left panel). Mass spectrum in negative mode (–MS) and product ion mass spectrum (–MS2) of *m/z* 363 (right panels), Figure S4: Screening of a library of family 1 UGTs containing enzymes from diverse plant species with (±)-menthol by whole-cell biotransformation. The strain that produced the highest amount of the glucoside product was set to 100%, Figure S5: Glucosylation of (±)-menthol catalyzed by UGT93Y1 (A) and UGT93Y2 (B). Ion trace *m/z* 363 [M + HCOO]^−^ is shown, Figure S6: Disorder prediction graph for the 3D model of UGT93Y1, Figure S7: Local UGT93Y1 model quality plot.

## Sample Availability

Samples of the compounds are not available from the authors.
