# Supplementary material for: Glucosylation of (±)-Menthol by Uridine-Diphosphate-Sugar Dependent Glucosyltransferases from Plants
Source: Molecules. 2021 Sep 10;26(18):5511. doi: 10.3390/molecules26185511 (PMC8470988; doi:10.3390/molecules26185511)
Supplement: Supplementary file 1 [file molecules-26-05511-s001.zip › molecules-1346413-supplementary.pdf]

Supplementary Materials:

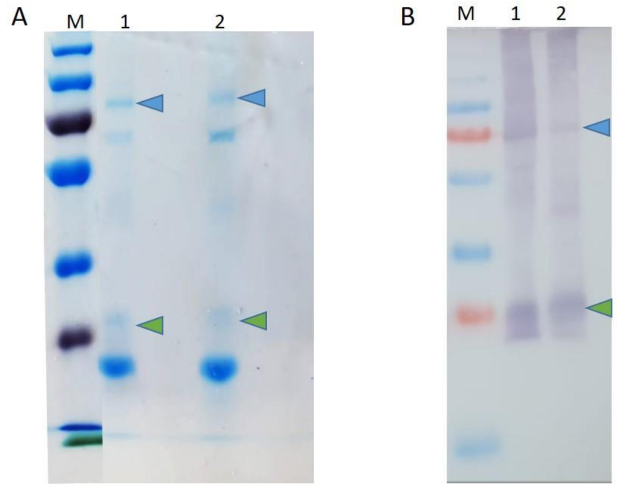

Figure S1. SDS-PAGE (**A**) and Western blot (**B**) using a primary monoclonal Anti-Glutathione-S-Transferase (GST) antibody produced in mouse (Sigma Aldrich) of recombinant UGT93Y1 (1) and UGT93Y2 (2) in purified protein elution fractions. Blue and green arrows show the UGTs and GST proteins, respectively. Marker proteins (M).

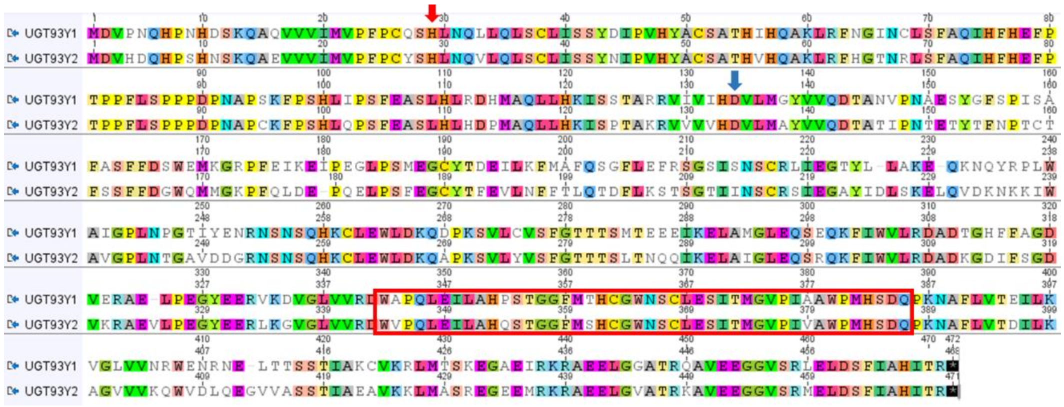

Figure S2. Amino acid sequences of UGT93Y1 and UGT93Y2 from *C. sinensis* var. *sinensis*. The catalytically active His and the activating Asp are marked with red and blue arrows, respectively. The red box represents the conserved PSPG box.

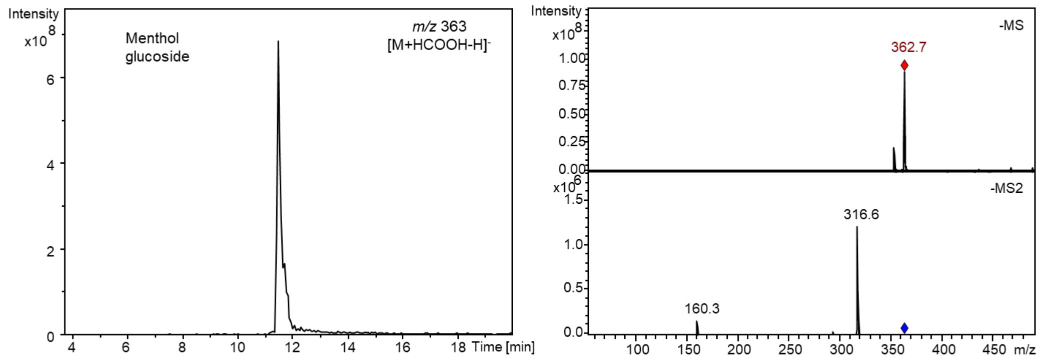

Figure S3. LC-MS analysis of (±)-menthyl glucoside Extracted ion chromatogram *m/z* 363 [M+HCOO]- (left panel). Mass spectrum in negative mode (–MS) and product ion mass spectrum (–MS2) of *m/z* 363 (right panels).

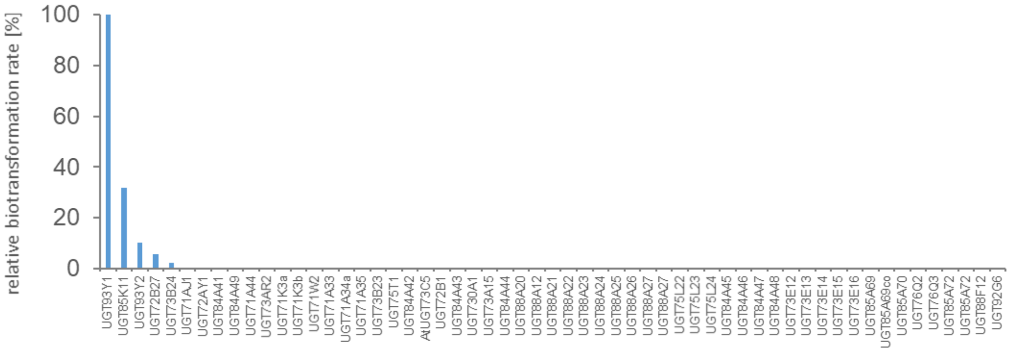

Figure S4. Screening of a library of family 1 UGTs containing enzymes from diverse plant species with (±)-menthol by whole-cell biotransformation. The strain that produced the highest amount of the glucoside product was set to 100%.

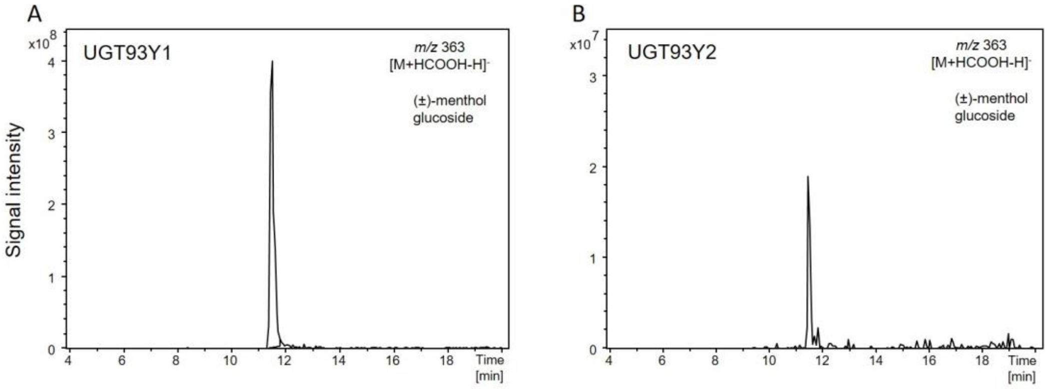

Figure S5. Glucosylation of (±)-menthol catalyzed by UGT93Y1 (**A**) and UGT93Y2 (**B**). Ion trace *m/z* 363 $[M+HCOO]^-$ is shown.

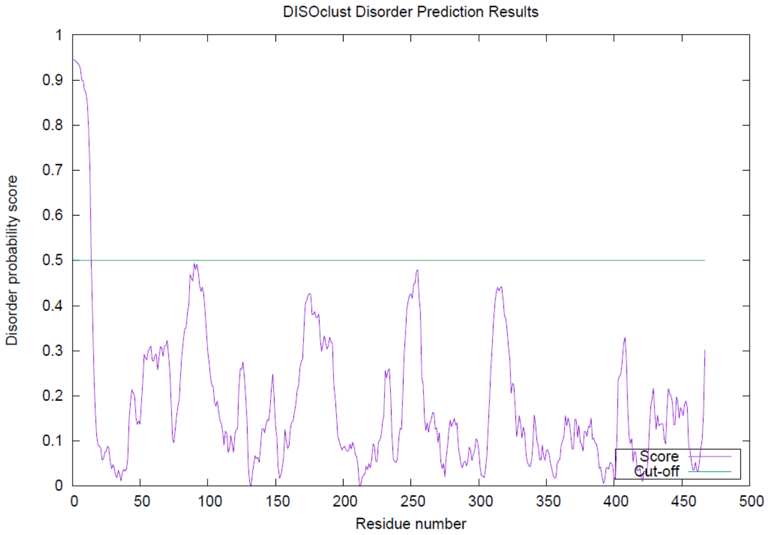

Figure S6. Disorder prediction graph for the 3D model of UGT93Y1. Calculated according to [1].

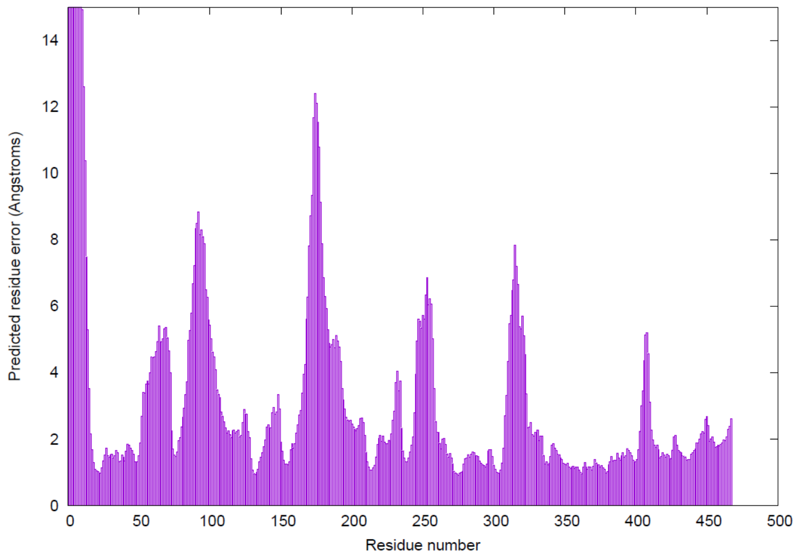

Figure S7. Local UGT93Y1 model quality plot [1].

1. McGuffin, L.J.; Adiyaman, R.; Maghrabi, A.H.A.; Shuid, A.N.; Brackenridge, D.A.; Nealon, J.O.; Philomina, L.S. IntFOLD: an integrated web resource for high performance protein structure and function prediction. *Nucleic Acids Research* **2019**, *47*, W408-W413, doi:10.1093/nar/gkz322.
